# Supplementary material for: A qPCR technology for direct quantification of methylation in untreated DNA
Source: Nat Commun. 2023 Aug 24;14:5153. doi: 10.1038/s41467-023-40873-y (PMC10449789; doi:10.1038/s41467-023-40873-y)
Supplement: Supplementary file 1 — Supplementary Information [file 41467_2023_40873_MOESM1_ESM.pdf]

Supplementary Information

Table 1. Mean melting temperatures and standard deviation in °C for targets and probes.

	Ref	INA-1	INA-2
UM	64.56 ± 0.03	72.38 ± 0.01	71.85 ± 0.25
1mC	67.46 ± 0.08	75.17 ± 0.05	77.52 ± 0.13
2mC	66.90 ± 0.05	74.83 ± 0.08	77.14 ± 0.12
3mC	66.63 ± 0.13	75.29 ± 0.06	77.2 ± 0.22
4mC	67.37 ± 0.06	75.28 ± 0.03	77.48 ± 0.19
1,2mC	68.19 ± 0.05	77.26 ± 0.12	79.64 ± 0.21
1,2,3mC	68.56 ± 0.05	79.72 ± 0.06	81.63 ± 0.09
1,2,3,4mC	68.89 ± 0.08	80.94 ± 0.14	82.8 ± 0.36

Table 2. Primers used in comparator method 1.

MGMT primers	Sequence 5’ to 3’-end
MGMT-M-FW	TTTCGACGTTCTAGGTTTTCGC
MGMT-M-REV	GCACTCTTCCGAAAACGAAACG
MGMT-U-FW	TTTGTGTTTTGATGTTTGTAGGTTTTTGT
MGMT-U-REV	AACTCCACACTCTTCCAAAAACAAAACA
