# Supplementary material for: A qPCR technology for direct quantification of methylation in untreated DNA
Source: Nat Commun. 2023 Aug 24;14:5153. doi: 10.1038/s41467-023-40873-y (PMC10449789; doi:10.1038/s41467-023-40873-y)
Supplement: Supplementary file 3 — Reporting Summary [file 41467_2023_40873_MOESM3_ESM.pdf]

nature portfolio

Corresponding author(s): Kamilla Kolding Bendixen

Last updated by author(s): Jul 19, 2023

Reporting Summary

Nature Portfolio wishes to improve the reproducibility of the work that we publish. This form provides structure for consistency and transparency in reporting. For further information on Nature Portfolio policies, see our Editorial Policies and the Editorial Policy Checklist.

Statistics

For all statistical analyses, confirm that the following items are present in the figure legend, table legend, main text, or Methods section.

n/a	Confirmed	
☐	☒	The exact sample size (*n*) for each experimental group/condition, given as a discrete number and unit of measurement
☐	☒	A statement on whether measurements were taken from distinct samples or whether the same sample was measured repeatedly
☒	☐	The statistical test(s) used AND whether they are one- or two-sided<br>*Only common tests should be described solely by name; describe more complex techniques in the Methods section.*
☒	☐	A description of all covariates tested
☐	☒	A description of any assumptions or corrections, such as tests of normality and adjustment for multiple comparisons
☐	☒	A full description of the statistical parameters including central tendency (e.g. means) or other basic estimates (e.g. regression coefficient) AND variation (e.g. standard deviation) or associated estimates of uncertainty (e.g. confidence intervals)
☒	☐	For null hypothesis testing, the test statistic (e.g. *F*, *t*, *r*) with confidence intervals, effect sizes, degrees of freedom and *P* value noted<br>*Give P values as exact values whenever suitable.*
☒	☐	For Bayesian analysis, information on the choice of priors and Markov chain Monte Carlo settings
☒	☐	For hierarchical and complex designs, identification of the appropriate level for tests and full reporting of outcomes
☒	☐	Estimates of effect sizes (e.g. Cohen's *d*, Pearson's *r*), indicating how they were calculated

Our web collection on statistics for biologists contains articles on many of the points above.

Software and code

Policy information about availability of computer code

Data collection	We used PCR software Real-time PCR system V1 belong to BaseTyper48.4 Quiet HRM Real-Time PCR System (PentaBase A/S, Odense, Denmark) and the CFX Maestro Software 2.0 belong to the CFX96 and CFX Opus 96 Real-time PCR instrument (Bio-Rad Laboratories, Inc., Hercules, CA, US) for data collection.
Data analysis	The following R-packages were used; cutpointr (1.1.2), dplyr (1.1.0), ggplot2 (3.4.1), ggmisc (0.5.2), ggpubr (0.6.0), readxl (1.4.2), tidyr (1.3.0), VennDiagram (1.7.3). All data analysis were first performed using used R studio version 1.3.1093 and R version 4.0.3, but no changes were found when updating to R studio version 2022.12.0 RStudio and R version 4.2.2.

For manuscripts utilizing custom algorithms or software that are central to the research but not yet described in published literature, software must be made available to editors and reviewers. We strongly encourage code deposition in a community repository (e.g. GitHub). See the Nature Portfolio guidelines for submitting code & software for further information.

Data

Policy information about availability of data

All manuscripts must include a data availability statement. This statement should provide the following information, where applicable:

- Accession codes, unique identifiers, or web links for publicly available datasets
- A description of any restrictions on data availability
- For clinical datasets or third party data, please ensure that the statement adheres to our policy

We have provided source data with the paper.

Human research participants

Policy information about studies involving human research participants and Sex and Gender in Research.

Reporting on sex and gender	Sex and gender did not have any impact on our study. Furthermore, the cohort is rather small, which would make it statistical challenging to make stratified analysis of the sex and gender. We have only included the information regarding the sex to describe the cohort used in the validation study.
Population characteristics	The cohort contained primarily glioblastoma samples (83%) and 57.1% of the samples were from men. The age at the time of biopsy spanned from 25 to 79 years with an average of 60.2 years. 88.1% was IDH1 wild type. Our cohort is represented by consecutive cases as we included all the patients diagnosed for a glioblastoma in our region.
Recruitment	The tumor sections used for the study were left-over samples. Inclusion criteria were: Age ≥ 18 years, histology-proven brain tumor (glioblastoma, oligodendroglioma, or astrocytoma), and availability of leftover tumor tissue or DNA for the validation of the assay. Exclusion criteria were: Age < 18 years and insufficient amount of leftover tissue material or extracted DNA.
Ethics oversight	Ethical approval code Ref. EC 3721 - BASEC 2020-01939 (16th October 2020) with ethics oversights by Comitato etico cantonale, Via Orico 5, 6501 Bellinzona CH

Note that full information on the approval of the study protocol must also be provided in the manuscript.

Field-specific reporting

Please select the one below that is the best fit for your research. If you are not sure, read the appropriate sections before making your selection.

☒ Life sciences ☐ Behavioural & social sciences ☐ Ecological, evolutionary & environmental sciences

For a reference copy of the document with all sections, see nature.com/documents/nr-reporting-summary-flat.pdf

Life sciences study design

All studies must disclose on these points even when the disclosure is negative.

Sample size	We did not make any sample-size calculations, as we only had access to 50 left-over samples. The scope of this study was to elaborate the technical details of the novel PCR-method, and to evaluate the method on a smaller cohort of clinical samples.
Data exclusions	We evaluated the concentration and volume available of the left-over samples. Eight samples were excluded as they did not contain sufficient material for analysis by all three methods used in the study.
Replication	We replicated the result of the EpiDirect® MGMT Methylation qPCR Assay of ten clinical samples at two different laboratories to confirm the results. We found that the results in terms of methylation status were identical. Data is not shown in the manuscript.
Randomization	Not relevant to this study as we have not divided the samples into different experimental groups.
Blinding	The samples were first analyzed using comparator method 1. The investigators were blinded to the methylation status of comparator method 1 when performing the analysis of comparator method 2 and EpiDirect® MGMT Methylation qPCR Assay. The final comparison with the methylation status evaluated using the three methods was done at the end, after the analysis of comparator methods 1 and 2.

Reporting for specific materials, systems and methods

We require information from authors about some types of materials, experimental systems and methods used in many studies. Here, indicate whether each material, system or method listed is relevant to your study. If you are not sure if a list item applies to your research, read the appropriate section before selecting a response.

Materials & experimental systems

n/a	Involved in the study
☒	☐ Antibodies
☒	☐ Eukaryotic cell lines
☒	☐ Palaeontology and archaeology
☒	☐ Animals and other organisms
☒	☐ Clinical data
☒	☐ Dual use research of concern

Methods

n/a	Involved in the study
☒	☐ ChIP-seq
☒	☐ Flow cytometry
☒	☐ MRI-based neuroimaging
